# Diesel exhaust particles increase nasal symptoms and IL-17A in house dust mite-induced allergic mice

**DOI:** 10.1038/s41598-021-94673-9

**Published:** 2021-08-11

**Authors:** Hahn Jin Jung, Young-Kyung Ko, Woo Sub Shim, Hyun Jik Kim, Dong-Young Kim, Chae-Seo Rhee, Moo Kyun Park, Doo Hee Han

**Affiliations:** 1 Department of Otorhinolaryngology-Head and Neck Surgery, Chungbuk National University College of Medicine, Chungbuk National University Hospital, Cheongju, Korea; 2 Graduate School of Medicine, Seoul National University College of Medicine, Seoul, Korea; 3 Department of Otorhinolaryngology-Head and Neck Surgery, Seoul National University College of Medicine, Seoul National University Hospital, 101 Daehak-ro, Jongno-gu, Seoul, 03080 Korea; 4 Sensory Organ Research Institute, Seoul National University Medical Research Center, Seoul, Korea

**Keywords:** Experimental models of disease, Respiratory tract diseases

## Abstract

Diesel exhaust particles (DEPs), traffic-related air pollutants, are considered environmental factors adversely affecting allergic diseases. However, the immunological basis for the adjuvant effects of DEP in allergic rhinitis (AR) remains unclear. Therefore, this study aimed to investigate the effect of DEP exposure on AR using a mouse model. BALB/c mice sensitized to house dust mite (HDM) were intranasally challenged with HDM in the presence and absence of DEP. Allergic symptom scores, serum total and HDM-specific immunoglobulins (Igs), eosinophil infiltration in the nasal mucosa, cytological profiles in bronchoalveolar lavage fluid (BALF), and cytokine levels in the nasal mucosa and spleen cell culture were analyzed. Mice co-exposed to HDM and DEP showed increased allergic symptom scores compared with mice exposed to HDM alone. Reduced total IgE and HDM-specific IgE and IgG1 levels, decreased eosinophil infiltration in the nasal mucosa, and increased proportion of neutrophils in BALF were found in mice co-exposed to HDM and DEP. Interleukin (IL)-17A level was found to be increased in the nasal mucosa of the co-exposure group compared with that in the HDM-exposed group. The levels of IL-4, IL-13, interferon-γ, IL-25, IL-33, and TSLP expression showed no difference between the groups with and without DEP treatment. Increased expression of IL-17A in the nasal mucosa may contribute to DEP-mediated exacerbation of AR in HDM-sensitized murine AR model.

## Introduction

The worldwide incidence of allergic diseases such as asthma, atopic dermatitis, and allergic rhinitis (AR) has gradually increased in recent years^1^. Current increase in allergy prevalence is associated with air pollution^2,3^. Among various types of air pollution, traffic-related air pollutants such as diesel exhaust particles (DEPs) are strongly linked to the development and exacerbation of allergic diseases^4^.

DEPs are a key source of the particulates contributing to ambient air pollution in urban areas, produced from diesel engines and are nearly omnipresent in the environment^5^. DEP is a carbon-based particulate material containing various transition metals and organic compounds^6^. Epidemiological and observational studies have revealed that exposure to DEPs increases the risk for allergic diseases^7–9^, and even increases the morbidity and daily mortality^10,11^. Experimental studies in animal models have also reported that an adjuvant effect of DEP is positively related with the exacerbation of respiratory diseases^12,13^.

However, the exact mechanisms underlying the involvement of DEP in AR exacerbation is still poorly understood. Therefore, this study aimed to investigate the effect of DEP in AR using the house dust mite (HDM)-sensitized AR mouse model. The present study investigated the exacerbation of AR symptoms and the increase in expression of inflammatory cytokines caused by exposure to DEP in AR mice.

## Results

### Symptom scores

**Figure 1:**
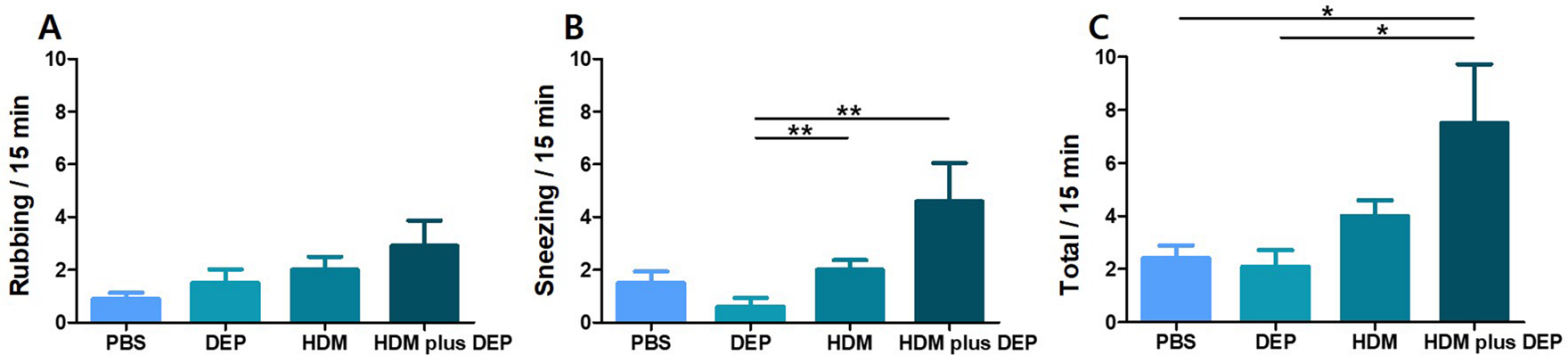
Symptom scores. (**A**) Nasal rubbing, (**B**) sneezing, and (**C**) total symptom score of each group. Data are expressed as mean ± standard error mean. *p < 0.05, **p < 0.01.

Figure 1 shows the symptom scores for each group after the last nasal challenge. The mice in the HDM and DEP co-exposed group showed higher allergic symptom scores for rubbing and sneezing, and higher total scores than mice exposed to HDM alone; however, the differences were not found to be statistically significant (all p value > 0.05). The mice in the HDM and DEP co-exposed group showed statistically higher total allergic symptom scores than those in the phosphate-buffered saline (PBS) and DEP-exposed groups (p value = 0.023 and 0.011, respectively). The mice exposed only to DEP showed no increase in allergic symptoms.

### Serum total and HDM-specific immunoglobulins

**Figure 2:**
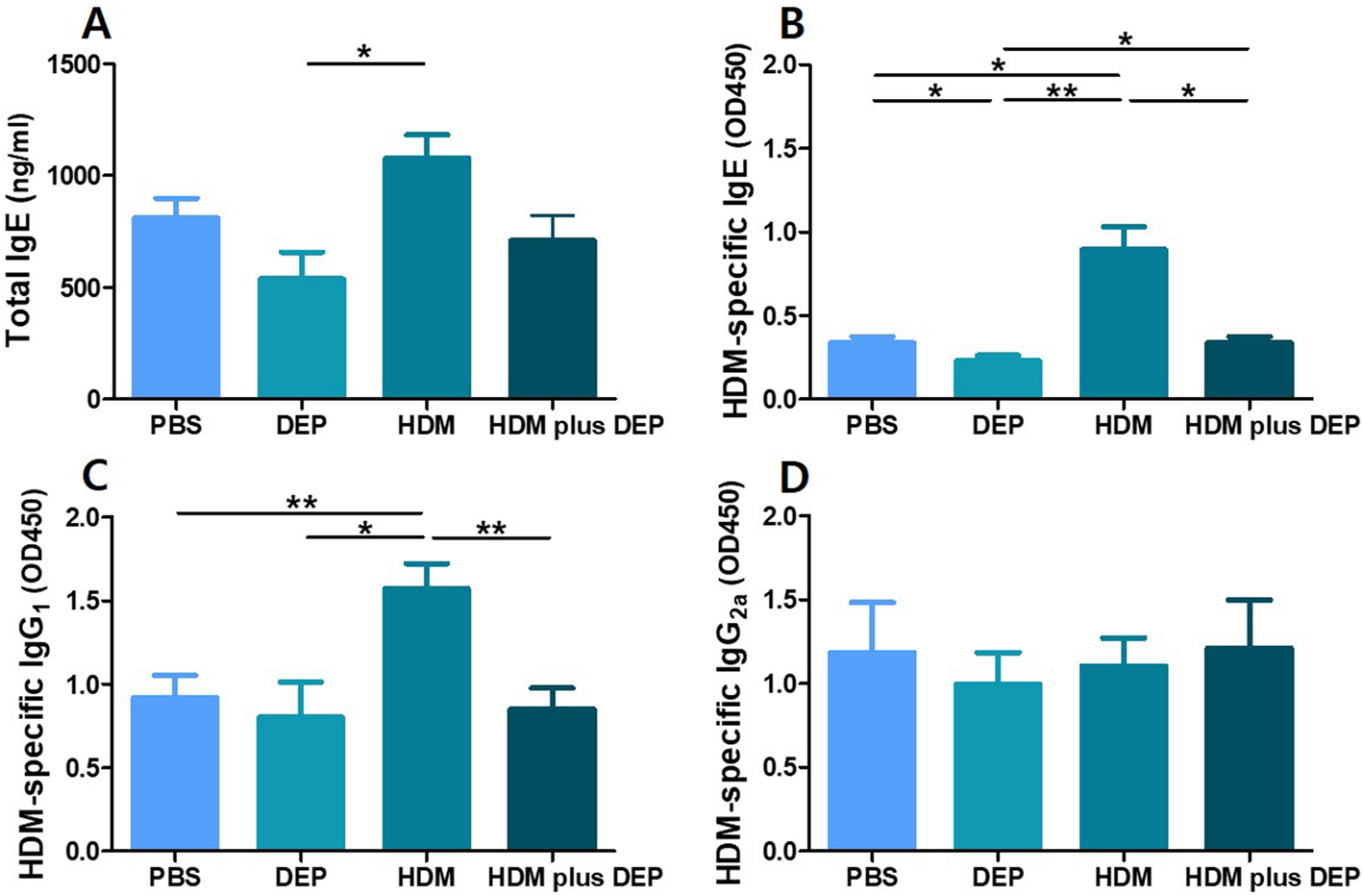
Serum immunoglobulin levels in each group. (**A**) Total IgE, (**B**) HDM-specific IgE, (**C**) HDM-specific IgG1, and (**D**) HDM-specific IgG2a. HDM and DEP co-exposed group showing suppressed levels of HDM-specific IgE and IgG_1_. Data are expressed as mean ± standard error mean. *p < 0.05, **p < 0.01.

The serum levels of total immunoglobulin (Ig) E and HDM-specific IgE and IgG1 (T_H_2-related Ig) in the HDM-exposed group showed significant elevation compared with those in the PBS and DEP-exposed groups (Fig. 2). However, the serum levels of HDM-specific IgE and IgG1 in the HDM and DEP co-exposed group showed significant decrease compared with those in the HDM-exposed group (p value = 0.015 and 0.009, respectively). The serum levels of HDM-specific IgG2a (Th1-related Ig) showed no difference between the groups.

### Eosinophil infiltration in the nasal mucosa

**Figure 3:**
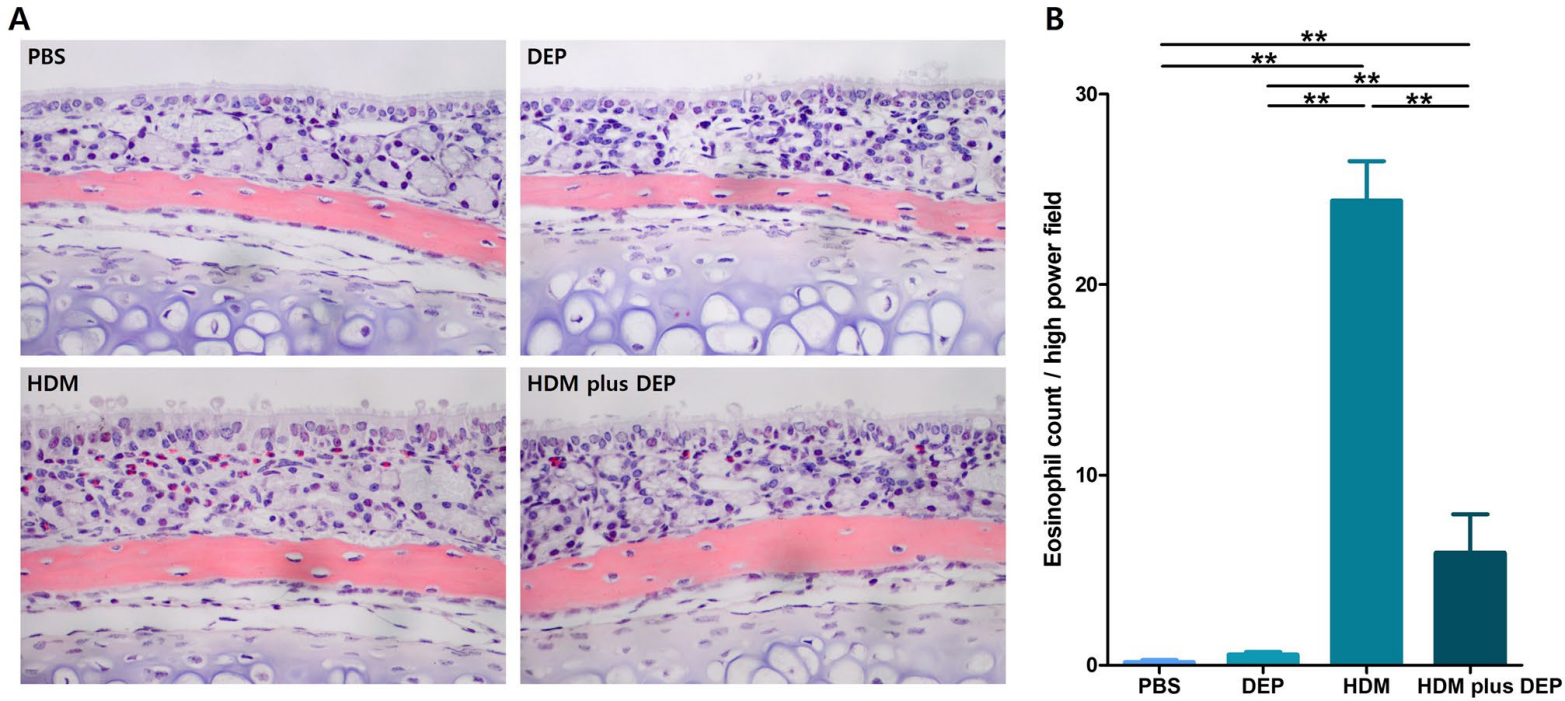
Eosinophil infiltration in the nasal mucosa. Co-exposure with HDM and DEP (group D) showing suppressed eosinophil infiltration in the nasal mucosa (**A**) Sirius red staining (× 400 magnification) of each group. Photographs are representative of nasal mucosa in each group. (**B**) Eosinophil count of the septal mucosa in each group. Data are expressed as mean ± standard error mean. *p < 0.05, **p < 0.01.

Figure 3 shows the number of eosinophils infiltrating the nasal mucosa per high-magnification field for each group. The HDM-exposed group and the HDM and DEP co-exposed group showed significantly elevated eosinophil counts compared with those in the PBS and DEP-exposed groups. However, there was a significant decrease in the eosinophil infiltration of the HDM and DEP co-exposure group compared with the HDM-exposed group (p value = 0.008).

### The cellular profiles in bronchoalveolar lavage fluid

**Figure 4:**
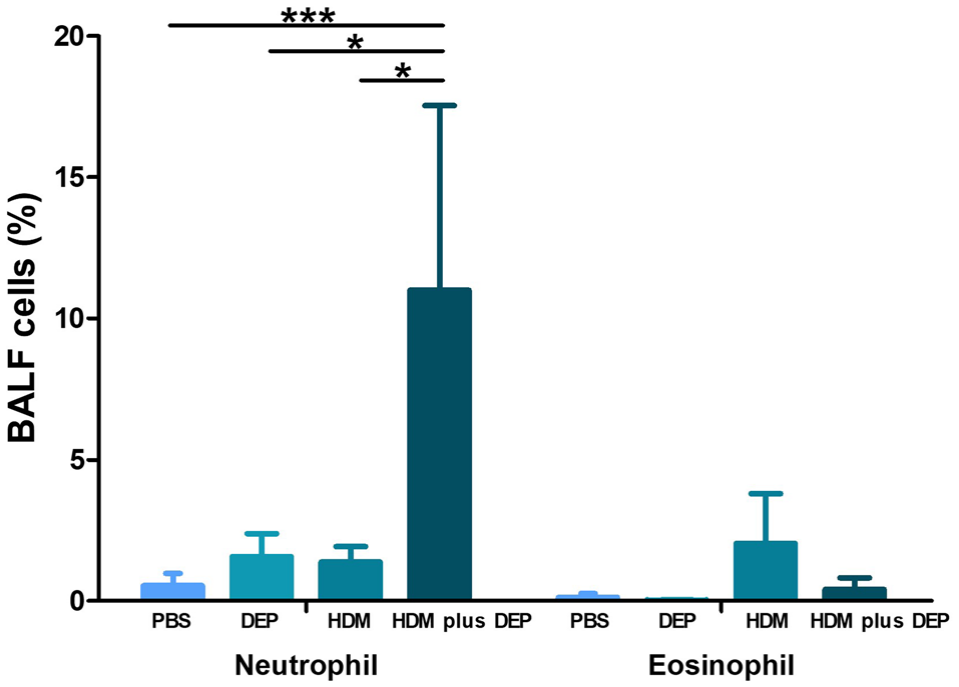
Differential counts in BALF. Significantly increased proportion of neutrophils in the BALF is seen in the HDM and DEP co-exposed group (group D). *p < 0.05, ***p < 0.001.

Figure 4 shows the effect of DEP treatment on the cellular profiles in bronchoalveolar lavage fluid (BALF). The HDM and DEP co-exposure group showed a significant increase in the proportion of neutrophils in the BALF as compared with the PBS, DEP, and HDM-exposed groups (p value ≤ 0.001, 0.028, and 0.015, respectively). The proportion of eosinophils in the BALF showed no difference between the groups.

### Cytokines in nasal mucosa

**Figure 5:**
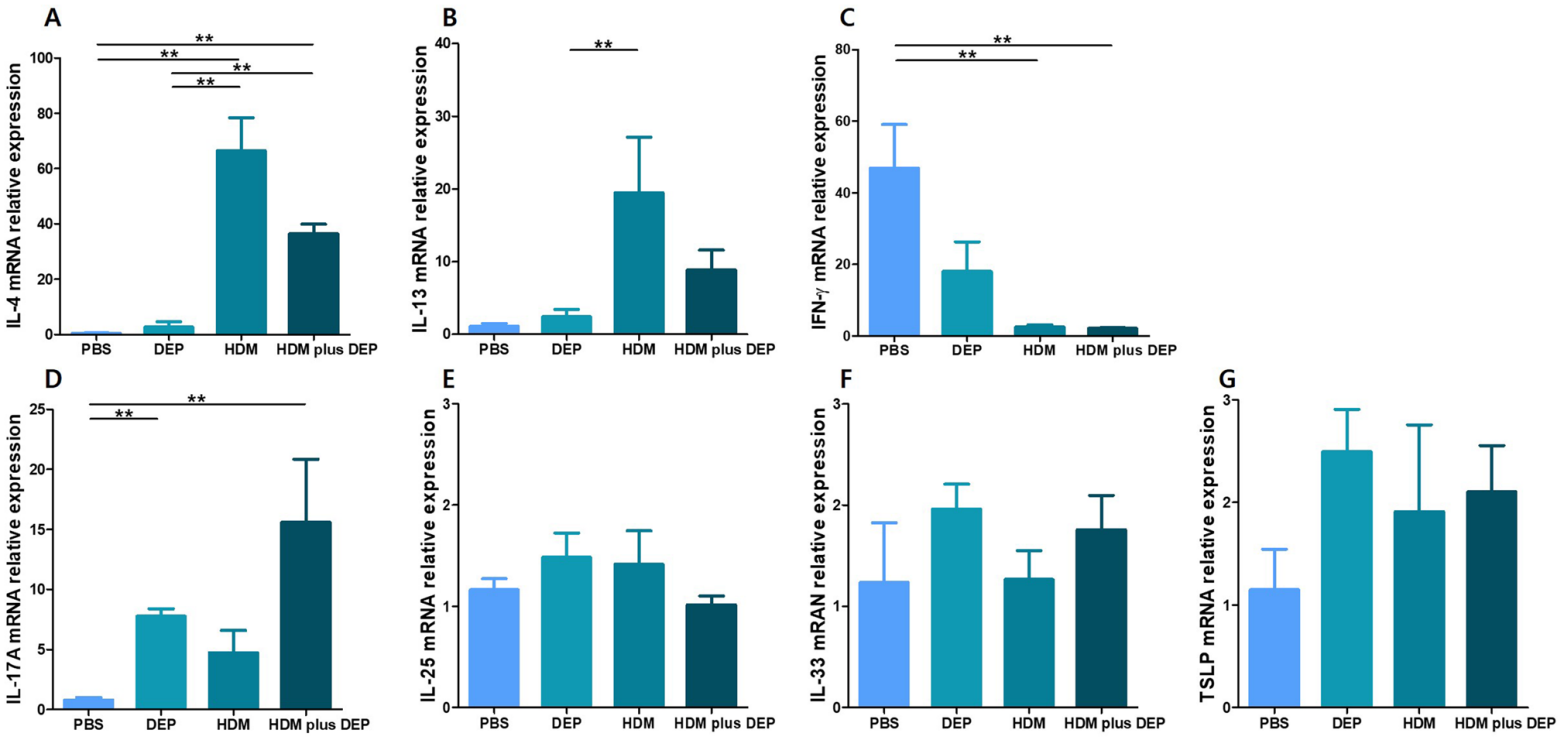
Real-time PCR analysis of cytokines expression in the nasal mucosa. Expression of (**A**) IL-4, (**B**) IL-13, (**C**) IFN-γ, and (**D**) IL-17A, (**E**) IL- 25, (**F**) IL-33, (**G**) TSLP in the nasal mucosa detected by real-time PCR. The transcriptional levels of IL-17A were increased in mice co-exposed to HDM and DEP. Data are expressed as mean ± standard error mean. **p < 0.01.

The mRNA levels of the inflammatory cytokines (Fig. 5) in the nasal mucosa were determined. Although the IL-4 showed significantly higher levels in the HDM-exposed group and the HDM and DEP co-exposed group as compared with the PBS and DEP-exposed groups, the HDM and DEP co-exposed group showed lower levels than the HDM-exposed group. However, the difference was statistically insignificant (p value = 0.151). IL-13 showed higher levels in the HDM-exposed group compared to the HDM and DEP co-exposed group; however, the difference was not significant (p value = 0.310). The mRNA levels of IFN-γ decreased in the HDM-exposed and the HDM and DEP co-exposed group. The concentration of IL-17A showed significantly higher levels in the DEP-exposed and the HDM and DEP co-exposed groups as compared with the PBS group (all p value = 0.008). In addition, the concentration of IL-17A were higher in the HDM and DEP co-exposed mice group compared to mice exposed only to HDM, which, however, did not reach statistical significance (p value = 0.056). The mRNA levels of IL-25, IL-33, and TSLP in the nasal mucosa showed no significant differences between the groups.

### Cytokines in spleen cell culture

**Figure 6:**
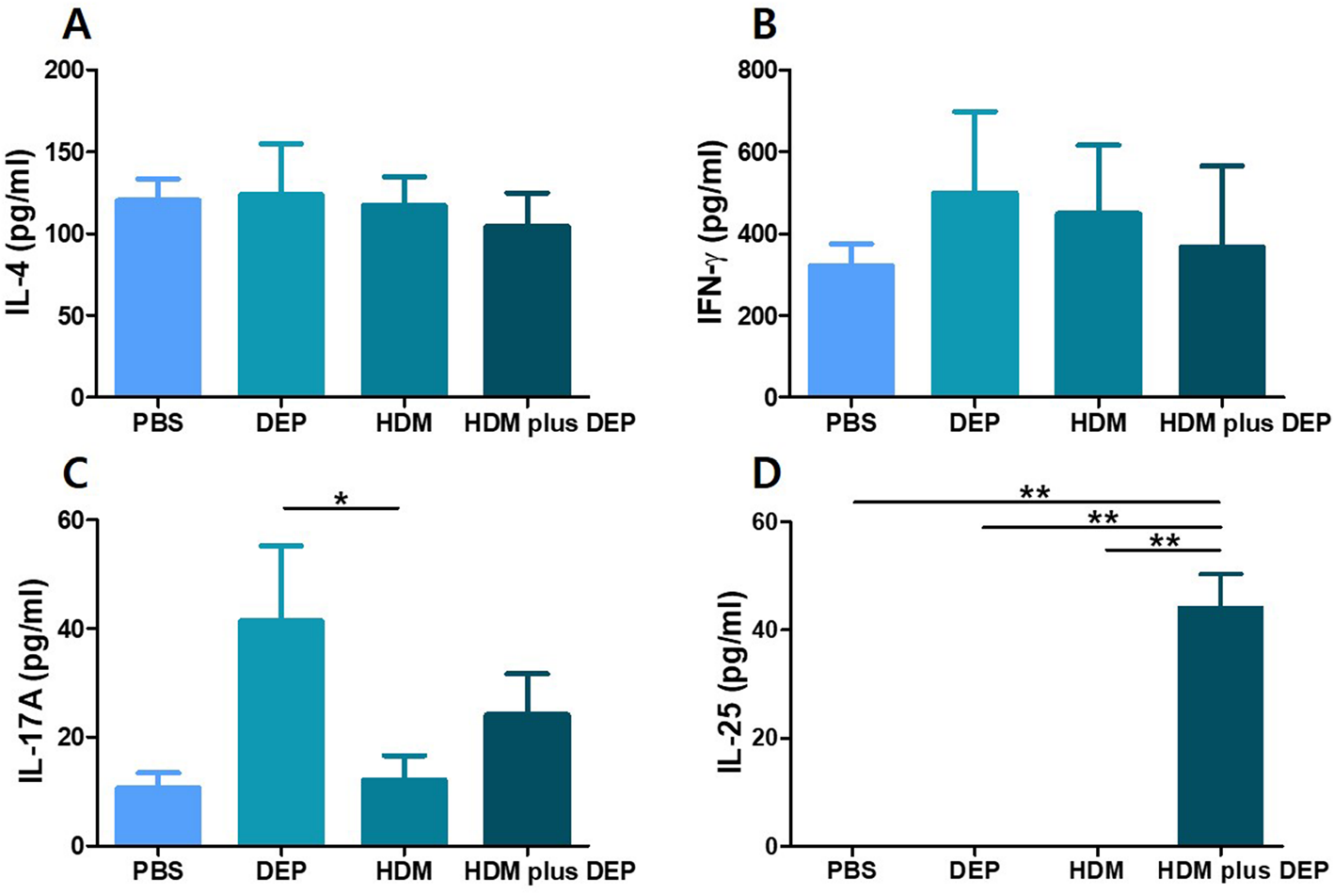
Measurement of cytokines in the spleen cell culture by ELISA. Concentration of (**A**) IL-4, (**B**) IFN-γ, (**C**) IL-17A, and (**D**) IL-25 in spleen cell culture of each group detected by ELISA. IL-25 was significantly increased in mice co-exposed to HDM and DEP. Data are expressed as mean ± standard error mean. *p < 0.05, **p < 0.01.

Figure 6 shows the levels of systemic cytokines from spleen cell culture. The levels of IL-4 and IFN-γ showed no difference among the groups. IL-17A level was significantly increased in the DEP-exposed group compared with that in the HDM-exposed group (p value = 0.032). However, there was no difference found between the HDM-exposed group and the HDM and DEP co-exposed group (p value = 0.421). Interestingly, splenocytes from mice co-exposed to HDM and DEP demonstrated a significantly higher level of IL-25 secretion compared with mice exposed only to PBS, DEP, and HDM (all p values = 0.008).

## Discussion

This study evaluated the effect of co-exposure to DEP, a major airborne fine particulate pollutant, on the HDM-sensitized AR mouse model. DEP co-exposure was associated with increased AR symptoms. Histological analysis showed that eosinophil infiltration was decreased in the nasal tissue and the neutrophil infiltration was increased in the BALF, upon co-exposure to DEP. Furthermore, increased IL-17A levels in nasal mucosa and IL-25 levels in the spleen cell culture were identified with concomitant exposure to DEP and HDM.

Recently, epidemiological evidence has extensively linked exposure to DEPs with development, progression, and exacerbation of AR^14–16^. The association between air pollution and increased risk of allergic disease might contribute to the dramatic increase in the incidence of allergic airway disease in industrialized countries^17–19^. The Cincinnati Childhood Allergy and Air Pollutions Study, a longitudinal prospective study, investigated DEP exposure in children and reported an increased prevalence of wheezing in the exposed children^20^. Furthermore, concomitant DEP and HDM exposure in murine models have been proven to enhance allergic responses^21–23^. Consistent with previous studies, this study showed that co-exposure to HDM and DEP exacerbated allergic symptoms. In addition, the daily symptoms observed in the HDM and DEP co-exposed group showed higher scores than the HDM-exposed group on all days, starting from the first day of the nasal challenge (data not shown).

However, the immunological mechanisms by which DEPs contribute to the exacerbation of AR remain poorly understood. A number of biological mechanisms have been proposed, including enhancement of T_H_2 inflammation, endotoxin-mediated damage, free radical production, oxidative stress, and the covalent modification of important cellular biomolecules^24^. Previous studies have indicated that co-exposure to an allergen and DEP could enhance T_H_2 responses with upregulated IgE production and eosinophilia^21,23^. Brandt et al. reported that repeated co-exposure to both HDM and DEP induced a significantly stronger effector T_H_2 response, characterized by increased BALF levels of T_H_2 cytokines and eosinophils^15^. Furthermore, excess production of reactive oxygen species (ROS) caused by inhalation of air pollutants has been shown to induce oxidative stress and result in allergic response^25–27^. Fukuoka et al. reported that DEP treatment disrupts tight junctions and impairs barrier function in the nasal epithelial cells; thus, individuals exposed to DEP become predisposed to allergen-induced rhinitis^22^.

Recently, the discovery of Th17 cells has added additional layers of complexity to the older T_H_1/T_H_2 balance paradigm of AR pathogenesis^28^. IL-17A, a key cytokine of T_H_17 cells, is a proinflammatory cytokine, and is known to induce tissue neutrophilia^29–31^. IL-17A and Th17 cells have been associated with neutrophilic inflammation and more severe asthma phenotypes, and have been correlated with the exacerbation of inflammation and the severity of airway hyperresponsiveness^32–34^. Brandt et al. have demonstrated that IL-17A contributes to asthma exacerbation and that co-exposure to HDM and DEP increases IL-17A producing T-cells^35^. They also reported that children with allergic asthma when exposed to high levels of DEP had nearly six times higher levels of serum IL-17A compared with children exposed to low levels of DEP^35^. The present study demonstrated that co-exposure to HDM and DEP significantly increased IL-17A level in nasal mucosa compared with mice exposed to HDM alone. In addition, the decreased eosinophil infiltration seen in histological analysis of nasal mucosa, and increased proportion of neutrophils seen in BALF analysis can be explained by increased IL-17A levels in nasal mucosa.

In the present study, systemic cytokines obtained via spleen cell culture showed increased levels of IL-25 (also known as IL-17E) upon DEP co-exposure. Recently, IL-25 was reported to have critical roles in promoting T_H_2-mediated inflammation^36–38^. IL-25 seems to increase in cultured splenocytes, since local DEP stimulation was added to an existing HDM-induced AR mouse model formed through the intraperitoneal and nasal challenge in our study. However, the increased secretion of IL-25 and the specific role of IL-25 in DEP-treated HDM-sensitized AR mice should be explored by further research.

The present study have several inherent limitations. First, even though the murine model is well-established, it cannot fully reproduce human AR. Second, it is proposed that the amount and type of allergen are important when evaluating the effect of DEP on AR. The relationship is complex and depends on the timing and duration of the exposure, as well as the dose and co-exposures^39^. The present study developed a mouse model with exposure to a clinically relevant allergen, HDM, and DEPs. HDM is one of the major inhalant allergens, and some studies have estimated that 10–20% of the population of any given country is allergic to HDM^40–42^. Further detailed studies are required to clarify the effect of DEP on AR using different allergens and various conditions of AR. Third, the immunological data were slightly T_H_2 prone in the PBS and DEP groups, since all mice were sensitized to HDM using intraperitoneal injections on days 0 and 7. However, under this study’s more identically controlled conditions, it could show more specific effects of DEP. Finally, BALF analysis suggested the possibility of an association between Th17 and increased neutrophil proportion; however, there was no evidence of neutrophils in the nasal tissue.

In conclusion, this study investigated the effect of DEP on HDM-sensitized AR in a mouse model. Particularly interesting finding was the increased expression of IL-17A in the nasal tissues instead of T_H_2 pathway. These findings can provide a new insight into the relationship between DEP and HDM-induced AR.

## Methods

### Mice

Six-week-old female BALB/c mice, weighing 16–19 g, were bred in pathogen-free conditions. All animal experiments in the present study followed the guidelines and ethics of the Institutional Animal Care and Use Committee (IACUC) of the Biomedical Research Institute of Seoul National University Hospital and were approved by the committee (IACUC number: 19-0014-S1A0). The study was carried out in compliance with the ARRIVE guidelines.

### Induction of allergic rhinitis in the murine model

**Figure 7:**
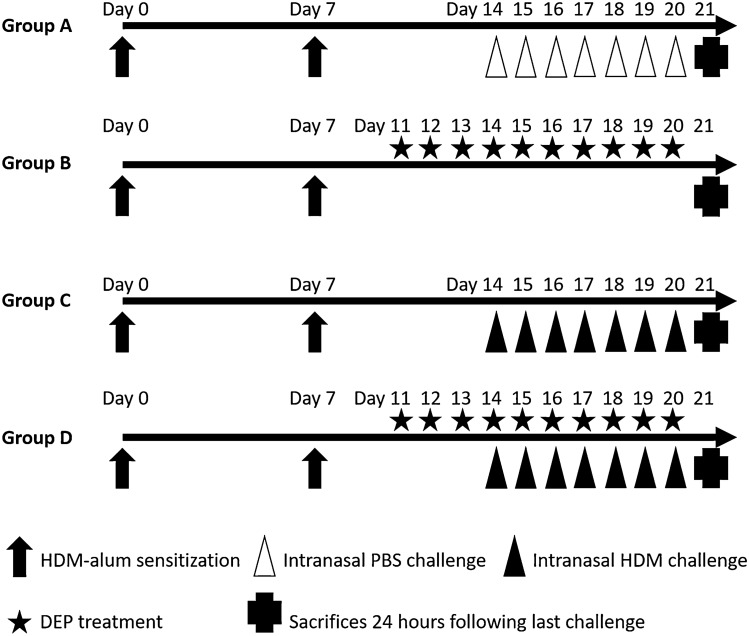
Experimental protocol. BALB/c mice were sensitized with intraperitoneal injection of HDM and alum on days 0 and 7. The mice in group A were intranasally challenged with PBS for seven consecutive days from day 14 to day 20. The mice exposed to DEP alone (group B) were treated with nasal instillation of DEP on days 11–20 without HDM. The mice in group C were intranasally challenged with HDM on days 14–20. The mice co-exposed to HDM and DEP (group D) were treated intranasally with DEP on days 11–20 and HDM on days 14–20 (2 h after administration of DEP). On day 20, the frequencies of nasal rubbing and sneezing behaviors were recorded for 15 min after the last intranasal challenge. Mice were sacrificed 24 h after the final challenge.

The mice were divided into four groups, each consisting of 10 mice, as follows: Group A, PBS-exposed group; Group B, DEP-exposed group; Group C, HDM-exposed group; and Group D, HDM and DEP co-exposed group. Allergen sensitization and challenge for the development of HDM-sensitized AR murine model are summarized in Fig. 7. Briefly, the mice were sensitized with an intraperitoneal injection of 100 μg HDM (Stallergenes Greer, Antony, France; #XPB82D3A25, D. pteronyssinus) complexed with 2 mg of aluminum hydroxide (alum) in 300 μL of PBS on days 0 and 7. The mice were then given 10 μg HDM extract (composed of 3.4 μg of protein, 0.086 μg of Der p 1, and 121.8 EU of endotoxin) in 20 μL of 0.05% Tween 80-PBS intranasally for 7 consecutive days starting day 14 to day 20. The mice in the PBS-exposed group were injected with HDM intraperitoneally and were intranasally challenged with 20 μL of 0.05% Tween 80-PBS instead of HDM on the same schedule. After sensitization with HDM, the mice in DEP-exposed group were intranasally administered with 100 μg DEP (National Institute of Standards & Technology, Gaithersburg, MD, USA; standard reference material 2975) suspended in 20 μL of 0.05% Tween 80-PBS on days 11–20 without HDM. The mice in the HDM and DEP co-exposed group were administered with DEP intranasally on days 11–20, and with HDM on days 14–20. HDM was given 2 h after administration of DEP on days 14–20.

### Symptom scores

The frequencies of nasal rubbing and sneezing behaviors were recorded by blinded observers during a 15-min period to evaluate early allergic responses on day 20, after the last intranasal challenge in each group. Total symptom score was calculated as the sum of nasal rubbing and sneezing. Mice were then sacrificed 24 h after the last challenge.

### Serum levels of total and HDM-specific immunoglobulins

Serum samples were collected from mice at the time of sacrifice. Serum samples were stored at − 80 °C until further use. Serum levels of total IgE and HDM-specific IgE, IgG_1_, and IgG_2a_ were measured by enzyme-linked immunosorbent assay (ELISA) as described previously^43,44^.

The total IgE levels were analyzed by coating the plates overnight with anti-mouse IgE capture monoclonal antibody (mAb) (BD PharMingen, San Jose, CA, USA) at 4 °C. The plate was washed with PBST (PBS containing 0.05% Tween-20) three times and nonspecific antigen–antibody reactions were blocked by incubation with 300 μL of 3% bovine serum albumin (BSA) per well for 1 h at room temperature. Serum samples were added to the 96-well plates along with purified mouse IgE isotype (BD PharMingen) used as a standard, and the plates were incubated for 3 h at 4 °C. The total IgE was detected by adding anti-mouse IgE-horseradish peroxidase (HRP) (#1130-055, Southern Biotechnology, Birmingham, AL, USA) to the plates. The HDM-specific IgE, IgG_1_, and IgG_2a_ were analyzed by coating the plates with 100 μg/mL HDM at 4 °C overnight. The plates were washed three times with PBST, and nonspecific antigen–antibody reactions were blocked by incubation with 300 μL of 3% BSA per well for 1 h at room temperature. Serum samples were added to the plates and incubated for 2 h at room temperature. The HDM- specific IgE, IgG_1_, and IgG_2a_ were detected by incubating each plate with biotin rat anti-mouse IgE, IgG_1_, and IgG_2a_ (BD PharMingen, San Jose, CA, USA). The plates were then washed three times and incubated with Streptavidin-HRP (BD PharMingen) for 30 min at room temperature. The reactions were developed using 3,3′,5,5′-tetramethylbenzidine (TMB) peroxidase substrate (Seracare life science, Milllford, MA, USA) and terminated by adding 1 M HCl. Optical density (OD) was measured in a microplate reader at 450 nm.

### Evaluation of histological findings in the nasal mucosa

The nasal histology of the mice was evaluated by fixing the heads of five mice in each group with 10% formaldehyde solution. The nasal tissues were decalcified with ethylenediaminetetraacetic acid (EDTA) solution, embedded in paraffin, sectioned at 4 μm thickness, and stained with Sirius red to visualize eosinophils. The infiltrating eosinophils were counted in five fields of the nasal septal mucosa by a single-blinded observer under a light microscope (400× magnification). Eosinophils were morphologically defined by the presence of eosinophilic granules in the cytoplasm and the presence of a two-lobed nucleus^45^.

### Analysis of bronchoalveolar lavage fluid

The effect of DEP on the HDM-induced recruitment of inflammatory cells was evaluated by measuring the proportion of macrophages, lymphocytes, neutrophils, eosinophils, and basophils in the BALF. The trachea was exposed after the sacrifice on day 21 and was cannulated with a 22-gauge catheter. The lungs were lavaged with 1 mL PBS, and the BALF was collected. The BALF was centrifuged at 1800 rpm at 4 °C for 10 min and the pellets were resuspended in PBS to measure the differential cell counts in the BALF. The differential cell count was determined by centrifuging the BALF at 1000 rpm at room temperature for 5 min using cyto-centrifugation (Cytopro 7620, Wescor, Logan, UT, USA), followed by staining using a Diff-Quik stain (Sysmex, Chuo-ku, Kobe, Japan), and fixation on slides. The relative numbers of different types of leukocytes were determined from the slides using a cell counter. Data were expressed as the ratio of the specific cell number to the total cell number.

### Real-time polymerase chain reaction for cytokines in the nasal mucosa

The nasal cavity was removed and the nasal mucosa of five mice in each group was carefully taken out using a curette. Total RNA was extracted from the nasal mucosa by a TRIzol LS reagent (Invitrogen, Carlsbad, CA, USA). Complementary DNA (cDNA) was synthesized using amfiRivert Platinum cDNA Synthesis Master Mix (GenDEPOT, Katy, TX, USA). cDNA for interleukin (IL)-4, interferon-gamma (IFN-γ), IL-17A, IL-25 (IL-17E), and glyceraldehyde-3-phosphate dehydrogenase (GAPDH) were amplified using QuantStudio 3 (Applied Biosystems, Foster City, CA, USA) in conjunction with TaqMan Fast advanced Master Mix (Applied Biosystems). The probes used were as follows: IL-4 (Mm 00445259_m1), IL-13 (Mm 00434204_m1), IFN-γ (Mm 99999071_m1), IL-17A (Mm 00439618_m1), IL-25 (Mm 00499822_m1), IL-33 (Mm 00505403_m1), TSLP (Mm 00498739_m1), and GAPDH (Mm 99999915_g1).

### Measurement of cytokines in the spleen cell culture

Cytokine ELISA was performed as previously described^44,46^. Briefly, spleen single-cell suspensions were plated in 24-well cell culture plates at a final concentration of 5 × 10^6^ cells/well using RPMI 1640 containing 10% fetal bovine serum (FBS) supplemented with 100 units/mL penicillin and 100 μg/mL streptomycin (Gibco, Grand Island, NY, USA). The cells were incubated in a CO_2_ incubator at 37 °C for 72 h and stimulated with HDM for 72 h. The culture supernatant was collected and stored at − 80 °C until cytokines were measured. Cytokine levels in the culture supernatant were assayed using a DuoSet ELISA kit (R&D Systems) according to the manufacturer’s protocol. The concentrations of IL-4, IFN-γ, IL-17A, and IL-25 were determined by interpolation from a standard curve after measuring the OD at 450 nm, and the data were expressed in pg/mL.

### Statistical analysis

The data are presented as means ± standard error of the mean (SEM). The statistical analyses used in this study include the Kruskal–Wallis test and the Mann–Whitney U test with the 2-tailed test for unpaired comparisons. Kruskal–Wallis tests were used to establish significant intergroup variability when comparisons were made between the groups. Mann–Whitney U tests (2-tailed) were then used for between-group comparisons. Statistical significance was considered when p < 0.05 (*), p < 0.01 (**), and p < 0.001 (***). Error bars represent the SEM. Statistical analysis was performed using SPSS 18.0 software (SPSS Inc., Chicago, IL, USA).

## Data Availability

All the data generated and/or analyzed during the current study are included in this article and are available from the corresponding author on reasonable request.
